# Unsatisfactory reproducibility of interstitial inflammation scoring in allograft kidney biopsy

**DOI:** 10.1038/s41598-023-33908-3

**Published:** 2023-05-01

**Authors:** Shun-Chen Huang, Yi-Jia Lin, Mei-Chin Wen, Wei-Chou Lin, Pei-Wei Fang, Peir-In Liang, Hao-Wen Chuang, Hui-Ping Chien, Tai-Di Chen

**Affiliations:** 1 Department of Anatomic Pathology, Chang Gung Memorial Hospital Kaohsiung Branch, Kaohsiung, Taiwan; 2 Department of Pathology, Tri-service General Hospital, National Defense Medical Center, Taipei, Taiwan; 3 Department of Pathology, China Medical University Hsinchu Hospital, Hsinchu, Taiwan; 4 Department of Pathology, National Taiwan University Hospital, Taipei, Taiwan; 5 Department of Pathology, Fu Jen Catholic University Hospital, Fu Jen Catholic University, New Taipei City, Taiwan; 6 Department of Pathology, Kaohsiung Medical University Hospital, Kaohsiung Medical University, Kaohsiung, Taiwan; 7 Department of Pathology and Laboratory Medicine, Kaohsiung Veterans General Hospital, Kaohsiung, Taiwan; 8 Department of Pathology and Laboratory Medicine, Shin Kong Wu Ho-Su Memorial Hospital, Taipei, Taiwan; 9 Department of Anatomic Pathology, Chang Gung Memorial Hospital Linkou Main Branch, Taoyuan, Taiwan

**Keywords:** Kidney diseases, Nephrology

## Abstract

Interstitial inflammation scoring is incorporated into the Banff Classification of Renal Allograft Pathology and is essential for the diagnosis of T-cell mediated rejection. However, its reproducibility, including inter-rater and intra-rater reliabilities, has not been carefully investigated. In this study, eight renal pathologists from different hospitals independently scored 45 kidney allograft biopsies with varying extents of interstitial inflammation. Inter-rater reliabilities and intra-rater reliabilities were investigated by kappa statistics and conditional agreement probabilities. Individual pathologists’ scoring patterns were examined by chi-squared tests and proportions tests. The mean pairwise kappa values for inter-rater reliability were 0.27, 0.30, and 0.26 for the Banff i score, ti score, and i-IFTA, respectively. No rater pair performed consistently better or worse than others on all three scorings. After dichotomizing the scores into two groups (none/mild and moderate/severe inflammation), the averaged conditional agreements ranged from 47.1% to 50.0%. The distributions of the scores differed, but some pathologists persistently scored higher or lower than others. Given the important role of interstitial inflammation scoring in the diagnosis of T-cell mediated rejection, transplant practitioners should be aware of the possible clinical implications of the far-from-optimal reproducibility.

## Introduction

Biopsy is an important tool for monitoring kidney allografts, and the findings of kidney allograft biopsy can guide clinical management and provide prognostic information. The Banff Classification of Renal Allograft Pathology, first published in 1993, is widely applied to the diagnosis of kidney allograft rejection^1^, and the importance of interstitial inflammation has been emphasized since the establishment of the classification. According to the most recent Banff classification update^2^, the diagnosis of acute T-cell mediated rejection requires a minimum of moderate tubulitis and moderate interstitial inflammation in the non-scarred cortex. Similarly, the diagnosis of chronic active T-cell mediated rejection requires a minimum of moderate tubulitis, moderate total cortical inflammation, and moderate inflammation in the scarred cortex.

Reproducibility, quantified by inter-rater and intra-rater reliabilities, is one of the most important attributes of any classification or scoring/grading scheme. Given the importance of interstitial inflammation in the diagnosis of kidney allograft rejection, good reproducibility is essential. Many studies have already confirmed the therapeutic and prognostic relevance of the Banff classification^3–6^. However, publications on the reproducibility, especially the inter- and intra-rater reliabilities of interstitial inflammation scorings, are scarce^7–12^. The reliability coefficients reported in these publications are quite varied and range from 0.33 to 0.65. A general understanding of how well or poorly renal pathologists perform on interstitial inflammation scoring assessments is not well established, the reported reliabilities coefficients are obscured to transplant practitioners, and the reasons for good or poor reliability have not been investigated; furthermore, to what extent the variation in interstitial inflammation scorings may impact clinical practice is unknown.

To address the above issues, the current study was undertaken to investigate the inter-rater and intra-rater reliabilities of interstitial inflammation scoring according to the Banff classification. In addition to the traditional kappa statistic approach, conditional agreements were calculated to improve the transparency and increase the understandability of inter- and intra-rater reliabilities for nephrologists, transplant surgeons, and renal pathologists. The findings provided a comprehensive picture of the reproducibility of interstitial inflammation scoring and should call attention to its possible clinical implications.

## Results

### Inter-rater reliabilities of inflammation scorings were suboptimal and had wide ranges

**Figure 1:**
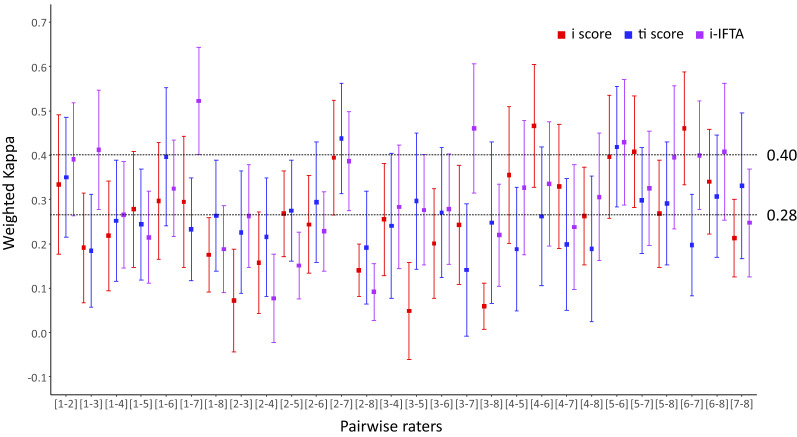
Pairwise inter-rater reliabilities of interstitial inflammation scorings grouped in rater pair order. The upper dashed line indicates a kappa value of 0.4, which is regarded as the lower limit of moderate agreement. The lower dashed line indicates the total average of all kappa values. Red, blue, and purple boxes and whiskers indicate the i score, ti score, and i-IFTA, respectively. Error bars: 95% confidence interval.

Table 1 summarizes the pairwise inter-rater reliabilities of interstitial inflammation scorings of the 8 raters (28 pairs in total). For inflammation in the non-scarred cortex (i score), pairwise weighted kappa values ranged from 0.14 (slight agreement) to 0.44 (moderate) and were 0.27 (fair) on average with a 95% confidence interval (CI) of 0.24 to 0.29. For total cortical inflammation (ti score), pairwise weighted kappa values ranged from 0.08 to 0.52. For inflammation in the scarred cortex (i-IFTA score), pairwise weighted kappa values ranged from 0.05 to 0.47. The inter-rater reliabilities of the three scorings did not significantly differ from each other (0.27 vs. 0.30 vs. 0.26, *P* = 0.147 by linear mixed model; LMM). Figure 1 shows the pairwise inter-rater reliabilities of interstitial inflammation scorings grouped in rater pair order. Most kappa values were lower than moderate agreement. No rater pair showed consistently better or worse inter-rater reliabilities on all three scorings, which was confirmed by one-way ANOVA (*P* = 0.063).

**Table 1:** Pairwise inter-rater reliabilities of interstitial inflammation scorings.

Weighted Kappa	i score (*n* = 28)	ti score (*n* = 28)	i-IFTA (*n* = 28)	*P*
Mean ± SD	0.27 ± 0.07	0.30 ± 0.11	0.26 ± 0.11	0.147
[Min, Max]	[0.14, 0.44]	[0.08, 0.52]	[0.05, 0.47]	
[95% CI]	[0.24, 0.29]	[0.26, 0.34]	[0.22, 0.31]

### Conditional agreement probabilities on scorings

Table 2 shows the conditional agreement probabilities for each rater on each inflammation score. For example, if rater 1 assigned i0 to a specific case, the probability that a random rater would also assign i0 to that case was 49.1%. Similarly, if rater 8 assigned ti0 to a specific case, the probability that a random rater would also assign ti0 to the case was 53.9%. Average conditional agreements ranged from 38.6% to 61.3% for the i score, 36.7% to 58.6% for the ti score, and 37.8% to 61.8% for the i-IFTA score. For many individual scores, chi-squared tests confirmed that raters performed differently (data not shown). However, no single rater had significantly better or worse agreement with other raters (*P* = 0.712; data not shown).

**Table 2:** Conditional agreement probabilities for each rater on each inflammation score.

Score/class	Raters (%, numerator/denominator)								Average	Mean ± SD
	Rater 1	Rater 2	Rater 3	Rater 4	Rater 5	Rater 6	Rater 7	Rater 8		
i score										
0	49.1 (212/432)	58.0 (153/264)	64.4 (103/160)	58.5 (117/200)	61.1 (176/288)	51.5 (202/392)	74.2 (95/128)	73.3 (88/120)	61.3%	61.3 ± 8.6
1	63.3 (81/128)	59.1 (137/232)	54.4 (248/456)	54.9 (211/384)	59.0 (236/400)	62.1 (154/248)	55.9 (210/376)	54.2 (269/496)	57.9%	57.9 ± 3.3
2	39.8 (35/88)	39.1 (50/128)	35.4 (34/96)	42.0 (37/88)	31.3 (10/32)	50.0 (28/56)	34.0 (68/200)	42.5 (34/80)	39.3%	39.3 ± 5.5
3	27.8 (20/72)	21.9 (21/96)	75.0 (6/8)	27.1 (13/48)	N/A	45.8 (11/24)	31.3 (5/16)	41.7 (10/24)	38.6%	38.6 ± 16.8
ti score										
0	63.8 (51/80)	65.6 (42/64)	70.8 (34/48)	44.4 (64/144)	53.5 (77/144)	41.5 (93/224)	72.5 (58/80)	53.9 (69/128)	58.3%	58.3 ± 10.9
1	62.1 (159/256)	59.9 (91/152)	61.1 (181/296)	57.3 (197/344)	55.2 (265/480)	55.8 (183/328)	65.2 (172/264)	52.2 (280/536)	58.6%	58.6 ± 4.0
2	37.5 (54/144)	25.0 (26/104)	33.9 (95/280)	38.6 (71/184)	50.0 (40/80)	36.5 (38/104)	34.7 (100/288)	37.5 (12/32)	36.7%	36.7 ± 6.4
3	37.1 (89/240)	28.8 (115/400)	55.2 (53/96)	41.7 (20/48)	81.3 (13/16)	65.6 (42/64)	54.5 (48/88)	66.7 (16/24)	53.8%	53.8 ± 16.3
i-IFTA										
0	56.0 (103/184)	65.6 (105/160)	N/A	76.0 (79/104)	58.3 (112/192)	65.6 (105/160)	65.8 (100/152)	45.1 (148/328)	61.8%	61.8 ± 9.0
1	53.6 (60/112)	62.5 (5/8)	39.8 (102/256)	44.0 (148/336)	43.5 (101/232)	44.1 (134/304)	54.2 (52/96)	36.9 (127/344)	47.3%	47.3 ± 8.0
2	38.8 (62/160)	30.4 (17/56)	34.2 (93/272)	38.9 (84/216)	40.4 (110/272)	39.5 (60/152)	36.5 (111/304)	43.8 (14/32)	37.8%	37.8 ± 3.8
3	36.7 (97/264)	30.6 (152/496)	45.8 (88/192)	53.1 (34/64)	79.2 (19/24)	55.8 (58/104)	50.6 (85/168)	81.3 (13/16)	54.1%	54.1 ± 17.0
Total, (%)										
Mean ± SD	47.1 ± 12.4	45.5 ± 17.6	51.8 ± 15.0	48.0 ± 12.7	55.7 ± 15.1	51.2 ± 10.0	52.4 ± 15.4	52.4 ± 14.4	–	
[Min, Max]	[27.8, 63.8]	[21.9, 65.6]	[33.9, 75.0]	[27.1, 76.0]	[31.3, 81.3]	[36.5, 65.6]	[31.3, 74.2]	[36.9, 81.3]	–	
[95% CI]	[39.3, 55.0]	[34.4, 56.7]	[41.8, 61.9]	[40.0, 56.1]	[45.6, 65.8]	[44.8, 57.5]	[42.7 , 62.2]	[43.3, 61.6]	–	

Given the critical interstitial inflammation thresholds for a definite diagnosis of acute T-cell mediated rejection (≥ i2) and chronic active T-cell mediated rejection (≥ ti2 and ≥ i-IFTA2), we dichotomized the scores into two groups: none/mild (score 0/1) and moderate/severe (score 2/3) inflammation (Table 3). Taking the top left cell (Rater 1; i score 0/1; 52.3%) for example, it indicates that in 52.3% of the time that a random rater would also assign i score 0 or 1 to those cases Rater 1 assigned i score 0 or 1. In other words, in 47.7% (1–52.3%) of the time that a random rater would disagree with Rater 1 and assigned those cases i score 2 or 3. The average conditional agreement for i score 0/1 was 57.2%, indicating that in 42.8% (1–57.2%) of the time, a second random rater would assign an i score of 2/3 to the case that the first rater considered i score 0/1. The average conditional agreements in general were 47.2% for i scores, 50.0% for ti scores, and 47.1% for i-IFTA scores. Therefore, the overall disagreement rate among all scorings was over 50%. Similar to the original 4-tier scorings, raters performed differently in some individual groups, but no single rater had significantly better or worse agreement with other raters (*P* = 0.983; data not shown).

**Table 3:** Conditional agreement probabilities for each rater on dichotomized scores.

Score/class	Raters (%, numerator/denominator)								Average	Mean ± SD
	Rater 1	Rater 2	Rater 3	Rater 4	Rater 5	Rater 6	Rater 7	Rater 8		
i score										
0/1	52.3 (293/560)	58.5 (290/496)	57.0 (351/616)	56.2 (328/584)	59.9 (412/688)	55.6 (356/640)	60.5 (305/504)	58.0 (357/616)	57.2%	57.2 ± 2.4
2/3	34.4 (55/160)	31.7 (71/224)	38.5 (40/104)	36.8 (50/136)	31.3 (10/32)	48.8 (39/80)	33.8 (73/216)	42.3 (44/104)	37.2%	37.2 ± 5.6
ti score										
0/1	62.5 (210/336)	61.6 (133/216)	62.5 (215/344)	53.5 (261/488)	54.8 (342/624)	50.0 (276/552)	66.9 (230/344)	52.6 (349/664)	58.0%	58.0 ± 5.7
2/3	37.2 (143/384)	28.0 (141/504)	39.4 (148/376)	39.2 (91/232)	55.2 (53/96)	47.6 (80/168)	39.4 (148/376)	50.0 (28/56)	42.0%	42.0 ± 8.0
i-IFTA										
0/1	55.1 (163/296)	65.5 (110/168)	39.8 (102/256)	51.6 (227/440)	50.2 (213/424)	51.5 (239/464)	61.3 (152/248)	40.9 (275/672)	52.0%	52.0 ± 8.3
2/3	37.5 (159/424)	30.6 (169/552)	39.0 (181/464)	42.1 (118/280)	43.6 (129/296)	46.1 (118/256)	41.5 (196/472)	56.3 (27/48)	42.1%	42.1 ± 6.9
Total, (%)										
Mean ± SD	46.5 ± 11.8	46.0 ± 17.4	46.0 ± 11.0	46.5 ± 8.1	49.2 ± 10.4	50.2 ± 3.5	50.7 ± 13.9	50.0 ± 7.1	–	
[Min, Max]	[34.0, 63.0]	[28.0, 65.0]	[38.0, 63.0]	[37.0 , 56.0]	[31.0 , 60.0]	[46.0 , 56.0]	[34.0 , 67.0]	[41.0 , 58.0]	–	
[95% CI]	[55.0, 63.0]	[62.0, 65.0]	[57.0, 63.0]	[53.0 , 56.0]	[55.0, 60.0]	[52.0 , 56.0]	[61.0, 67.0]	[56.0 , 58.0]	–	

### Intra-rater reliabilities were better than inter-rater reliabilities

Table 4 shows the descriptive statistics of intra-rater reliabilities and inter-rater reliabilities on three interstitial inflammation scorings. The intra-rater reliabilities were generally better than the inter-rater reliabilities (0.37 vs. 0.27, 0.49 vs. 0.30, and 0.44 vs. 0.26 for the i score, ti score, and i-IFTA score, respectively).

**Table 4:** Kappa statistics of intra-rater reliabilities and inter-rater reliabilities.

Variable	Intra-rater reliability (*n* = 8; Mean ± SD)	Inter-rater reliability (*n* = 28; Mean ± SD)
i score	0.37 ± 0.14	0.27 ± 0.07
ti score	0.49 ± 0.15	0.30 ± 0.11
i-IFTA	0.44 ± 0.15	0.26 ± 0.11

### Pathologists’ practicing patterns of scoring varied

**Figure 2:**
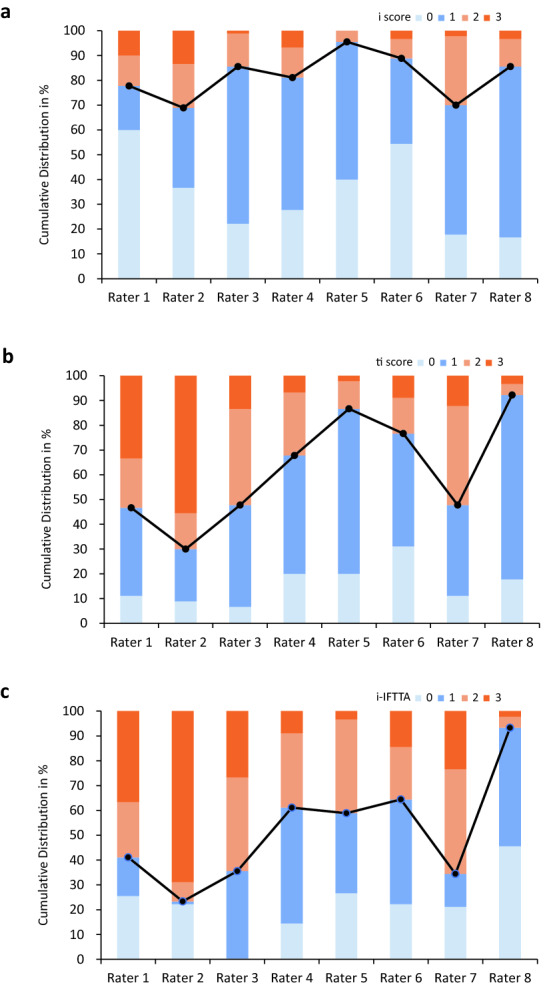
Distribution of scores assigned to the cases by each of the 8 raters. (**a**) i score. (**b**) ti score. (**c**) i-IFTA score. Note the similar pattern across i, ti, and i-IFTA scores on distributions of none/mild (0/1) vs. moderate/severe (2/3) groups, highlighted by broken lines.

Figure 2 shows the distribution of the scores assigned to the cases by each of the 8 raters. Raters displayed different tendencies on the extent of inflammation they assigned to cases, which was more noticeable in dichotomizing the scores into clinically relevant groups of none/mild (score 0/1) and moderate/severe (score 2/3) inflammation. For example, rater 5 tended to give lower scores, and rater 2 preferred higher scores. Interestingly, although the exact proportions varied, individual pathologist’s tendencies in scoring shared a common pattern across the i score, ti score, and i-IFTA. Chi-squared tests confirmed the differences in score distribution on both the 4-tier and 2-tier categorizations (*P* < 0.001; Supplementary Table 1 and 2). For each scoring, post hoc tests with Bonferroni correction confirmed that rater 5 consistently assigned lower scores compared to raters 2 and 7, and scores assigned by rater 6 were also significantly lower than those of rate 2.

## Discussion

Interstitial inflammation scoring (i, ti, and i-ITFA) is routinely carried out and is an essential part of the diagnostic criteria for T-cell mediated rejection in the Banff Classification of Renal Allograft Pathology. Given the important roles of the scorings, reproducibility is essential. However, most of the time, the reproducibility of the scoring has been overlooked, and good reproducibility is usually taken for granted. In contrast, in this study, we showed that the inter-rater reliability of interstitial inflammation scoring is not good or even remotely reasonable. We found that pairwise inter-rater reliabilities of interstitial inflammation scoring were only fair in general and varied widely, with kappa values ranging from simply not acceptable (0.05) to moderate at most (0.52). Pathologists perform no better or worse on any particular scoring, and individual pathologists do not perform significantly different from each other. Overall, inter-rater reliabilities of interstitial inflammation scoring were unsatisfactory and showed no identifiable patterns that we could use to predict the performance of any pair of raters.

Inflammation in the non-scarred cortex (i score) has been included in the Banff classification from its establishment^1^, and its inter-rater reliability in a few studies^7–11^ revealed suboptimal results (kappa or intraclass correlation coefficient from 0.26 to 0.42). Although cannot be compared directly, our findings on the Banff i score (mean weighted kappa 0.27) concurred with the above studies. Interstitial inflammation is routinely assessed in allograft kidney biopsies ae well as in native kidney biopsies. Therefore, the finding of poor inter-rater reliability is not only surprising but also alarming. The Banff classification continuously evolves according to newly found evidence, and the concepts of total cortical inflammation (ti score) and inflammation in the scarred cortex (i-IFTA score) were subsequently introduced in the 2007 and 2015 meetings^13,14^. These scores play important roles in the diagnosis of chronic active T-cell mediated rejection. The inter-rater reliability of ti score was investigated in a study on preimplantation biopsy, and the intraclass correlation coefficient was reported to be 0.44 (fair)^11^. A surprisingly high reliability of i-IFTA scores (pairwise kappa values 0.60 to 0.65) was reported by the Paris group^12^. This latter study included only three pathologists in the same study group, which might be the reason for the unusually high agreement. We found that the inter-rater reliabilities of the Banff ti score and i-IFTA were as unsatisfactory as those of the Banff i score. However, these findings should be validated in future studies.

The above studies used different reliability coefficients (Cohen’s kappa, Fleiss kappa, and intraclass correlation coefficient) to investigate inter-rater reliability. Although all the results except the one from the Paris group are equally considered suboptimal by standards^15,16^, it is hard to directly compare the findings to each other due to different coefficient constructs and different sample populations. It is also very important to bear in mind that intraclass correlation coefficient or kappa statistics depend on the scoring distributions to some extent when the agreement differs across different locations on the continuous explanatory variable scale or across different categories. Using kappa statistics to infer reliability in another population requires the scoring distribution of the sample cases similar to that of the population. On the other hand, conditional agreement probabilities, given that the sample is representative, can be compared and generalized to the population.

More importantly, unlike kappa statistics or intraclass correlation coefficients, which are obscured to most nephrologists, transplant surgeons, and renal pathologists, conditional agreement probabilities give us a clear view on how well or poorly raters agree with each other and represent what we will encounter in daily practice, expressed in percentages. For example, sentences like “There is a 57.2% chance that another pathologist won’t agree with you on the extent of interstitial inflammation in this case.” are more understandable than “The kappa statistic for the inter-rater reliability of interstitial inflammation is 0.27.” A similar approach has been proposed in the field of pathology^17^. As expected, in concordance with the kappa statistic values, we found pathologists did not agree with each other frequently on interstitial inflammation scoring. Pathologists perform equally poorly on all three kinds of scorings, and no single pathologist performed better than others. Even after dichotomizing scores into none/mild (score 0/1) and moderate/severe (score 2/3) inflammation groups, which is more clinically relevant, disagreement rates were still over 50%. Given that interstitial inflammation is a prerequisite for the diagnosis of T-cell mediated rejection, such poor agreement on scoring might result differences in pathological diagnosis and potentially impact clinical practice. Accordingly, future studies are warranted to investigate the effects of irreproducible pathological scorings on clinical decision.

By comparing intra-rater reliabilities with inter-rater reliabilities, we found that pathologists were much more consistent with themselves but could not reconcile with each other. An intriguing and illuminating finding, evidently on charts and statistically proven, is that in contrast to the generally poor inter-rater reliabilities, raters do have consistent intrinsic practice patterns of their own. The distributions of scores assigned by individual raters were different, but at the same time, a general intra-rater pattern was visually evident across the three inflammation scorings. Chi-squared tests with post hoc two proportions tests by Bonferroni’s correction confirmed the above observations, indicating pathologists do have their own tendencies in scoring the extent of interstitial inflammation, and these tendencies were the same for i, ti, and i-IFTA scoring. For example, rater 2 consistently assigned higher scores on all three scorings than other raters. This finding partially explained the poor inter-rater reliabilities across raters but at the same time, better intra-rater reliabilities within individuals. The fixed practice pattern could be a double-edged sword. On the one hand, we would be reassured by good internal consistency at a single institution where only one pathologist reads all the biopsies. On the other hand, scores across different centers and studies are unlikely to be comparable, and general practice in the real world might be hampered by the poor inter-rater reliabilities.

We concur with Marcussen et al. in their very first study on inter-rater reliability of the Banff classification that more precise criteria for the semiquantitative scores are needed^7^. The visual analog scales for Banff ci and ct scores provided in a recent reference guide might be an example^18^. However, it has also been shown by an international study that the inter-rater reliability of the Banff i score (along with many other histological features in kidney biopsy) does not improve after persistent feedback^10^. Therefore, potential benefits from educational actions also need to be evaluated in future works.

In conclusion, in concordance with previous studies, we confirmed that the inter-rater reliability of Banff i scoring is moderate at most. In addition, we found pathologists’ performance on inter-rater reliabilities of the Banff ti score and i-IFTA were equally poor. By conditional agreement probabilities, we clearly showed that on average, the agreement on interstitial inflammation scores between any two pathologists was no better than chance, and the poor inter-rater reliability was at least partially rooted in pathologists’ individual preferences when scoring the extent of interstitial inflammation. The findings of the current study should alert nephrologists, transplant surgeons, and pathologists of the uncertainty of inflammation scores as well as the Banff classification and encourage more investigation into the reasons and possible remedies for the poor inter-rater reliability.

## Methods

### Datasets

Kidney allograft biopsies between 2018 and 2020 performed at Linkou Chang Gung Memorial Hospital were used. Fifty cases representing the full spectrum of interstitial inflammation were selected from the archive by one senior renal pathologist (T.C.), who did not participate in the subsequent scoring process for the assessment of inter-rater and intra-rater reliabilities. The slides were scanned and converted to whole-slide images with a NanoZoomer S360 Digital slide scanner C13220-01 at 400X magnification. The study was approved by the Chang Gung Medical Foundation Institutional Review Board (IRB No.: 202200101B0). All experiments were performed in accordance with relevant guidelines and regulations. Written informed consent was waived by the Chang Gung Medical Foundation Institutional Review Board. The study adhered to the Declaration of Helsinki.

### Interstitial inflammation scoring by pathologists

Eight renal pathologists from 8 different hospitals in Taiwan carried out the scoring independently. All participating pathologists had been specially trained in the field of renal pathology for one or two years. The ages of the pathologists are from 32 to 64, and they have a wide range of independent sign-out experience in kidney allograft pathology from 2 to 24 years (mean = 7.6), working in different classes of institutions from primary district hospitals to tertiary medical centers. All pathologists were provided with whole-slide images of hematoxylin and eosin-, periodic acid-Schiff-, periodic acid-methenamine silver-, and trichrome-stained sections of each case and performed interstitial inflammation scoring according to their usual practice following the Banff classification^18^. The scorings were reported using the Banff classification for inflammation in the non-scarred cortex (i0: absent/minimal, < 10% of non-scarred cortex inflamed; i1: mild, 10–25%; i2: moderate, 26–50%; i3: severe, > 50%), total cortical inflammation (ti0: absent/minimal, < 10%; ti1: mild, 10–25%; ti2: moderate, 26–50%; ti3: severe, > 50%), and inflammation in the scarred cortex (i-IFTA0: absent/minimal, < 10% of non-scarred cortex inflamed OR if the extent of cortical IFTA is < 10%; i-IFTA1: mild, 10–25% of scarred cortex inflamed; i-IFTA2: moderate, 26%-50% of scarred cortex inflamed; i-IFTA3: severe, > 50% of scarred cortex inflamed). Five cases were excluded from the subsequent analysis due to missing scores from at least one rater. The remaining 45 cases were scored twice with an interval of at least three months for evaluation of intra-rater reliability. Because one rater might assign different scores during the first and second rounds, for evaluation of inter-rater reliability, scores from the first and second rounds were pooled together to get 90 scores that were used to obtain a balanced interpretation. For simplicity, we refer to the 90 scores as cases in the manuscript.

### Conditional agreement probabilities

Conditional agreement probabilities were calculated by observed concordant scores. For each case, there were 8 different scores assigned by 8 raters. The conditional agreement of a particular rater on a specific score was calculated by the counts of the specific score divided by counts of all scores assigned by all raters to cases that the particular rater assigned that specific score. For example, regarding inflammation of the non-scarred cortex (i score), 54 out of 90 cases were scored i0 by rater 1. From these 54 cases, we had 432 (8 raters × 54 cases) scores assigned by raters. Out of these 432 scores, there were 212 i0 scores (and 220 i1, i2, or i3 scores), so the conditional agreement for rater 1 on score i0 is 212 ÷ 432 = 49.1%, meaning that 49.1% of the time, a random rater would also assign i0 to any case scored i0 by rater 1. We see our raters as a representative sample of the entire renal pathologist population. Taking out one rater as the first rater for a case has a neglectable influence on the entire population. Therefore, the first rater’s scores were not removed from the numerator and denominator.

### Statistics

Inter-rater and intra-rater reliabilities were evaluated by pairwise linearly weighted Cohen’s kappa. The weighted kappa coefficients among different scorings (i, ti, and i-IFTA) were compared using a linear mixed model (LMM) which included two random effects: the intercept of the subject (the case) and the random slope of scoring. The LMM is able to deal with the dependency of the 28 pairwise kappa coefficients and 3 scorings. The distributions of scores among the eight raters were compared using chi-squared test. Comparisons between proportions were carried out by 2-proportion tests. Data analyses were conducted using SPSS 26 (IBM SPSS Inc, Chicago, Illinois). A *P* value less than 0.05 was considered statistically significant.

### Ethical approval

The studies involving human participants were reviewed and approved by Chang Gung Medical Foundation Institutional Review Board. Written informed consent was waived by the Chang Gung Medical Foundation Institutional Review Board.

## Supplementary Information

Supplementary Information.

## Data Availability

The datasets generated and/or analyzed during the current study are available from the corresponding author on reasonable request.
