# Supplementary material for: Unsatisfactory reproducibility of interstitial inflammation scoring in allograft kidney biopsy
Source: Sci Rep. 2023 May 1;13:7095. doi: 10.1038/s41598-023-33908-3 (PMC10151326; doi:10.1038/s41598-023-33908-3)
Supplement: Supplementary file 1 — Supplementary Information. [file 41598_2023_33908_MOESM1_ESM.pdf]

Supplementary material to:

Unsatisfactory reproducibility of interstitial inflammation scoring in allograft kidney biopsy

Shun-Chen Huang, Yi-Jia Lin, Mei-Chin Wen, Wei-Chou Lin, Pei-Wei Fang, Peir-In Liang, Hao-Wen Chuang, Hui-Ping Chien and Tai-Di Chen

Supplementary Table 1. Differences in score distribution on the 4-tier categorization.

	Rater 1 (*n* = 90)	Rater 2 (*n* = 90)	Rater 3 (*n* = 90)	Rater 4 (*n* = 90)	Rater 5 (*n* = 90)	Rater 6 (*n* = 90)	Rater 7 (*n* = 90)	Rater 8 (*n* = 90)	*P*
i score									<0.001
0	54 (60.0)	33 (36.7)	20 (22.2)	25 (27.8)	36 (40.0)	49 (54.4)	16 (17.8)	15 (16.7)	
1	16 (17.8)	29 (32.2)	57 (63.3)	48 (53.3)	50 (55.6)	31 (34.4)	47 (52.2)	62 (68.9)	
2	11 (12.2)	16 (17.8)	12 (13.3)	11 (12.2)	4 (4.4)	7 (7.8)	25 (27.8)	10 (11.1)	
3	9 (10.0)	12 (13.3)	1 (1.1)	6 (6.7)	0 (0.0)	3 (3.3)	2 (2.2)	3 (3.3)	
ti score									<0.001
0	10 (11.1)	8 (8.9)	6 (6.7)	18 (20.0)	18 (20.0)	28 (31.1)	10 (11.1)	16 (17.8)	
1	32 (35.6)	19 (21.1)	37 (41.1)	43 (47.8)	60 (66.7)	41 (45.6)	33 (36.7)	67 (74.4)	
2	18 (20.0)	13 (14.4)	35 (38.9)	23 (25.6)	10 (11.1)	13 (14.4)	36 (40.0)	4 (4.4)	
3	30 (33.3)	50 (55.6)	12 (13.3)	6 (6.7)	2 (2.2)	8 (8.9)	11 (12.2)	3 (3.3)	
i-IFTA									<0.001
0	23 (25.6)	20 (22.2)	0 (0.0)	13 (14.4)	24 (26.7)	20 (22.2)	19 (21.1)	41 (45.6)	
1	14 (15.6)	1 (1.1)	32 (35.6)	42 (46.7)	29 (32.2)	38 (42.2)	12 (13.3)	43 (47.8)	
2	20 (22.2)	7 (7.8)	34 (37.8)	27 (30.0)	34 (37.8)	19 (21.1)	38 (42.2)	4 (4.4)	
3	33 (36.7)	62 (68.9)	24 (26.7)	8 (8.9)	3 (3.3)	13 (14.4)	21 (23.3)	2 (2.2)	

Supplementary Table 2. Differences in score distribution on the 2-tier categorization.

	Rater 1 (*n* = 90)	Rater 2 (*n* = 90)	Rater 3 (*n* = 90)	Rater 4 (*n* = 90)	Rater 5 (*n* = 90)	Rater 6 (*n* = 90)	Rater 7 (*n* = 90)	Rater 8 (*n* = 90)	*P*
i score									<0.001
0-1	70 (77.8)	62 (68.9)	77 (85.6)	73 (81.1)	86 (95.6)[ab]	80 (88.9)[b]	63 (70.0)[ef]	77 (85.6)	
2-3	20 (22.2)	28 (31.1)	13 (14.4)	17 (18.9)	4 (4.4)	10 (11.1)	27 (30.0)	13 (14.4)	
ti score									<0.001
0-1	42 (46.7)	27 (30.0)	43 (47.8)	61 (67.8)b	78 (86.7)[abc]	69 (76.7)[abc]	43 (47.8)[ef]	83 (92.2)[abcdg]	
2-3	48 (53.3)	63 (70.0)	47 (52.2)	29 (32.2)	12 (13.3)	21 (23.3)	47 (52.2)	7 (7.8)	
i-IFTA									<0.001
0-1	37 (41.1)	21 (23.3)	32 (35.6)	55 (61.1)[bc]	53 (58.9)[bc]	58 (64.4)[abc]	31 (34.4)[def]	84 (93.3)[abcdefg]	
2-3	53 (58.9)	69 (76.7)	58 (64.4)	35 (38.9)	37 (41.1)	32 (35.6)	59 (65.6)	6 (6.7)	

a, b, c, d, e, f and g indicated significant different from rater 1, rater 2, rater 3, rater 4, rater 5, rater 6, rater 7 by two proportions tests with Bonferroni's correction.
